# Collaborative learning in the professional development of medical radiation practitioners

**DOI:** 10.1002/jmrs.548

**Published:** 2021-09-18

**Authors:** Michelle Turner, Sanya Morasi, Monica Mrsnik‐Hamdi, Madeleine Shanahan

**Affiliations:** ^1^ Discipline of Medical Radiation Science Faculty of Health University of Canberra Bruce Australian Capital Territory Australia

**Keywords:** Continuous professional development, education, radiation therapists, radiographer, social media

## Abstract

**Introduction:**

Medical radiation practitioners (MRPs) participate in continuous professional development (CPD) to update their knowledge, skills, safety standards and patient care. The Medical Radiation Practice Board of Australia (MRPBA) recommends that practitioners participate in a variety of activities and to incorporate the use of collaborative learning tools. The aim of this research was to investigate the value, use and workplace supports for online and face‐to‐face collaborative learning for CPD.

**Methods:**

A cross‐sectional online survey of Australian MRPs was conducted. The questionnaire was distributed via e‐blast from the Australian Society of Medical Imaging and Radiation Therapy (ASMIRT) to members.

**Results:**

A total of 115 completed questionnaires were received. Seminars, workshops and conferences were the most valued collaborative learning tools, with no significant difference in ranking observed (*P* > 0.05). The majority of MRPs regularly attend conferences (64%, *n* = 73) with those working in a metropolitan location more likely to attend. MRPs are supported by their workplace to attend conferences through the provision of paid leave (61%, *n* = 63), funding (50%, *n* = 52) and to a lesser extent travel expense (38%, *n* = 39). More than half (60%, *n* = 69) of the participants use social media for CPD with Facebook being the most frequently used and most useful online platform. The most common reasons for using social media for CPD were accessibility to information (85%, *n* = 56), little geographical limitations (77%, *n* = 51) and ease of use (74%, *n* = 49).

**Conclusion:**

Medical radiation practitioners currently utilise both face‐to‐face and online collaborative learning tools to meet their CPD needs. Face‐to‐face tools are more frequently utilised and highly valued by MRPs.

## Graphical abstract

To maintain registration with the Australian Health Practitioner Regulation Agency (AHPRA) medical radiation practitioners (MRPs) must participate in continuous professional development (CPD). Seminars, workshops and conferences were ranked by MRPs the most valued collaborative learning tools and no significant difference was indicated (*P* > 0.05).

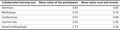

## Introduction

Medical radiation practitioners (MRPs) inclusive of radiographers and radiation therapists participate in continuous professional development (CPD) to update their knowledge, skills, competency, safety standards and improve their patient care.^1^, ^2^ To maintain registration with the Australian Health Practitioner Regulation Agency (AHPRA), MRPs must attain 60 h of CPD over a 3‐year period with a minimum of 10 h attained each year.^3^ The Medical Radiation Practice Board of Australia (MRPBA) recommends that practitioners participate in a variety of learning activities such as interactions with peers in the form of collaborative learning.^3^

Collaborative learning can be defined as a group of individuals coming together with various levels of knowledge to learn from and with others and work towards a common goal.^4^ Collaborative learning is highly beneficial and can be supported through face‐to‐face or online via social learning platforms.^4^ MRPs utilise and value a range of face‐to‐face collaborative learning opportunities, including conferences, seminars, workplace training and workshops to update their professional knowledge and skills.^5^, ^6^, ^7^ Although these are highly valued, practitioners require workplace support such as funding, time during work hours and access to the activities.^6^, ^8^, ^9^ Stevens and Wade^9^ identified that finding time for CPD can be difficult due to staff shortages and family commitments. Health practitioners working in rural or remote areas reported access to CPD activities was limited as travelling for seminars or conferences was a barrier.^8^, ^10^ As a result, there is interest in online and social media, to reduce learning barriers of geographic location and time.^11^

With advances in technology professional development, opportunities have expanded. MRPs have gained access to online platforms and information in the form of web pages, journal articles, online books, blogs, images and videos, as well as interactive platforms such as social media to meet their professional learning needs.^11^ Social media is a form of online communication technology that enables users to share information, knowledge and opinions with others in the community.^12^, ^13^, ^14^ A recent survey by the Australian Bureau of Statistics^15^ reported that 59 per cent of Australians access social media every day or most days, and more than a third access social media more than five times per day. Boyd et al.^11^ investigated the use of online media by MRPs and demonstrated that while social media was not widely adopted by MRPs to meet their professional development needs, increase in future use of these tools by MRPs was anticipated. As social media becomes increasingly prevalent,^14^, ^15^ it is important to establish how collaborative learning, both through social media and face to face, is currently valued and being adopted by MRPs to meet their professional learning needs.

The aim of this study was to better understand the current value and use of collaborative learning tools, including social media, in CPD activities of MRPs. In particular, this paper examined the following research questions.

1. What collaborative learning tools are most frequently utilised by MRPs for CPD?
2. What value is attributed to these tools? And does it vary by geographic workplace location?
3. How does the workplace support the use of collaborative learning tools for CPD?

## Material and Methods

The University Human Research Ethics Committee (2019‐2146) approved the project, including its design and recruitment methods.

### Study design

A cross‐sectional online survey design was utilised, with data collected through Qualtrics™ (Provo, UT, USA). The questionnaire was developed after a critical review of the literature, and pilot tested with four Australian MRPs to enhance clarity and coverage of content before it was operationalised. The questionnaire consisted of four key sections: (1) participant demographics, (2) use of collaborative learning tool including social media for CPD, (3) value of these tools for CPD and (4) workplace supports. The questions included 5‐point Likert scales for example usefulness (extremely useful [1] – not useful [5]) and value ranking (least important [1] to most important [5]).

Australian MRPs were invited to participate in the study, anonymously via online survey. The introduction to the survey provided participants with information outlining the aims, requirements and confidentiality of the study. Informed consent was obtained in the first item of the online survey, with respondents able to exit at this point if they so preferred. A link to the survey was distributed through an e‐blast to members of the Australian Society of Medical Imaging and Radiation Therapists (ASMIRT) in November 2019. As the link to the survey was electronic, further distribution of the survey link by e‐blast recipients may have occurred. Medical radiation students were excluded from the study as they are not required to undertake CPD.

### Sample size

Using the total population of individuals who held membership with ASMIRT (7054)^16^ as this was the primary survey distribution method, 95% confidence level, and a 5% margin of error, the total sample size of 365 was required.^17^ To determine whether a generally representative sample was achieved, demographic data of the sample were compared with registrant demographic data from the Australian Health Practitioner Regulation agency (AHPRA).^18^

### Data analysis

The data were uploaded to SPSS statistics (version 25.0. IBM Corp, Armonk, NY, USA). Descriptive and inferential statistics were used to analyse the data. Percentages were applied to describe the overall number of practitioners using collaborative learning for CPD and workplace support for CPD. The collected demographic data allowed for cross‐tabulations to determine whether associations existed across workplace demographics. Differences between groups were examined using chi‐square analysis, and where cell size was <5, Fisher’s exact test was utilised. A Friedman test was conducted on survey data to obtain an overall ranking of the importance MRPs attribute to collaborative learning tools for CPD, (1)‐for all participants and (2)‐for participants belonging to rural and remote workplaces as the literature identified rural and remote practitioners having reduced access to traditional face‐to‐face learning offerings. A p‐value less than 0.05 was the level for statistical significance used throughout the analysis.

## Results

### Demographics

This survey received 121 responses, of which 7 were incomplete, resulting in 115 completed and valid responses. Data on demographics of respondents are presented in Table 1. The majority of respondents were radiographers (76%) with 70% of the total respondents being females. Thirty per cent of participants were between the ages of 30–39 years, and 23% were under the age of 29 years. Survey participants were from metropolitan, regional, rural and remote workplace locations, with 62% of respondents working in the public health sector.

**Table 1:** Demographic characteristics of the participants (*n* = 115).

Characteristics	Frequency (%)
Gender	
Male	34 (30)
Female	81 (70)
Age	
Under 29 years	27 (23)
30–39 years	34 (30)
40–49 years	23 (20)
50–59 years	21 (18)
Above 60 years	10 (9)
Primary profession	
Radiographer	87 (76)
Radiation therapist	20 (17)
Nuclear medicine technologist	2 (2)
Sonographer	6 (5)
Primary employer	
Public health sector	71 (62)
Private health sector – large/corporate	32 (28)
Private health sector – small independent	10 (8)
University	2 (2)
Geographical location	
Metropolitan	72 (62)
Regional	17 (15)
Rural	23 (20)
Remote	3 (3)

### Collaborative learning tools for CPD

CPD activities undertaken in the workplace by MRPs are presented in Table 2. The most common activities reported were ‘Participating in training’ (90%, *n* = 103), ‘Online learning’ (71%, *n* = 82) and ‘Reading articles’ (71%, *n* = 82). Statistically significant differences for participation in CPD activities were observed across workplace demographics (Table 2, *P* < 0.05) for attendance at seminars, participation in online learning, provision of additional study time and attendance at journal clubs.

**Table 2:** Participation in CPD activities and relationship between participation in CPD activities in the workplace and workplace geographic location and sector.

**Workplace CPD**	**Geographic location (Metropolitan, regional, rural and remote)**			**Sector (Public/Private)**		
**Total (*n*, %)**	**Test of difference**	***P* value**	**Difference in experience of MRPs**	**Test of difference**	***P* value**	**Difference in experience of MRPs**
Attend seminar (*n* = 79, 69%)	7.390^‡^	*P* = 0.025*	78% (*n* = 56) of MRS professionals working in a metropolitan location attended workplace seminars for CPD compared with 53% (*n* = 9) working in regional location and 54% (*n* = 14) in a rural or remote location	7.649	*P* = 0.006**	71% (*n* = 55) of MRS professionals employed in the public sector attended a workplace seminar for CPD compared with 52% (*n* = 22) in private sector
Participate in training (*n* = 103, 90%)	0.353^†,a^	*P* > 0.99	89% (*n* = 64) of MRS professionals working in a metropolitan location participated in workplace training for CPD, similar to 88% (*n* = 15) working in regional location and 92% (*n* = 24) in a rural or remote location	0.085^‡^	*P* > 0.99	89% (*n* = 63) of MRS professionals employed in the public sector participated in workplace training for CPD similar to 91% (*n* = 38) in private sector
Read articles (*n* = 82, 71%)	5.931^†,a^	*P* = 0.057	89% (*n* = 23) of MRS professionals working in a rural or remote location read articles for CPD compared with 64% (*n* = 46) working in a metropolitan location and 77% (*n* = 13) in regional location	0.149^‡^	*P* = 0.699	70% (*n* = 50) of MRS professionals employed in the public sector read articles for CPD compared with 74% (*n* = 31) in private sector
Participate in online learning (*n* = 82, 71%)	9.024^†,a^	*P* = 0.012*	92% (*n* = 24) of MRS professionals working in a rural or remote location undertake online learning for CPD compared with 77% (*n* = 13), working in regional location and 63% (*n* = 45) in a metropolitan location	0.670‡	*P* = 0.413	69% (*n* = 49) of MRS professionals employed in the public sector undertake online learning for CPD compared with 76% (*n* = 32) in private sector
Attend journal club (*n* = 21, 18%)	3.457^†,a^	*P* = 0.182	24% (*n* = 17) of MRS professionals working in a metropolitan location attend journal club for CPD compared with 12% (*n* = 2), working in regional location and 8% (*n* = 2) a rural or remote location	10.768‡	*P* = 0.001**	27% (*n* = 19) of MRS professionals employed in the public sector have attend journal club for CPD compared with 2% (*n* = 1) in private sector
Additional study time (*n* = 20, 17%)	0.145^†,a^	*P* > 0.99	18% (*n* = 13) of MRS professionals working in a metropolitan location have additional study time for CPD, a finding similar to 18% (*n* = 3) working in regional location and 15% (*n* = 4) a rural or remote location	6.942^†^	*P* = 0.008**	24% (*n* = 17) of MRS professionals who work in the public sector have additional study time for CPD compared with 5% (*n* = 2) in private sector

^†^Fisher’s exact test, ^‡^chi‐square.

*P < 0.05, **P < 0.0.

^a^ Monte Carlo method used (95% CI) 10,000 sampled tables

Respondents were asked about their attendance at conferences. Most participants regularly attend conferences (64%, *n* = 73), with 36% (*n* = 41) attending more than once a year, and 28% (*n* = 32) attending one every two years (Table 3). MRPs working in a metropolitan area were most likely to attend a conference more than once a year (44%, *n* = 32) or one every two years (29%, *n* = 21).

**Table 3:** Participants conference attendance regularity (*n* = 115).

Conference attendance amount	Frequency (%)
More than once a year	41 (36%)
One every two years	32 (28%)
One every three years	7 (6%)
One every four years	2 (2%)
Less than one every four years	12 (10%)
I will be in the future	9 (8%)
Never	12 (10%)
Total number of participants	115

Frequency of use (Table 4) and usefulness for CPD (Table 5) of specific online platforms were explored. As demonstrated in Table 4, 60% (*n* = 69) of participants reported using social media for CPD. Facebook was the most frequently used platform for CPD with 20% (*n* = 10) using it daily and 23% (*n* = 12) at least once a week. Instagram and LinkedIn were also utilised for CPD purposes, primarily on a monthly basis. Across all listed social media platforms (Table 5), most participants consider the platforms not useful for CPD (selecting scale 4 or 5 on Likert scale). Of the platforms selected, Facebook (*n* = 19, 31%) and YouTube (*n* = 15, 28%) were considered the most useful social media platforms for CPD.

**Table 4:** Frequency of social media platforms used for CPD.

Social media platform access for CPD	At least daily	At least weekly	At least monthly	Total
	Frequency (%)	Frequency (%)	Frequency (%)	Frequency
Facebook	10 (20)	12 (23)	29 (57)	51
Instagram	4 (13)	4 (13)	22 (74)	30
Twitter	3 (11)	4 (14)	21 (75)	28
LinkedIn	3 (8)	5 (14)	28 (78)	36
ResearchGate	1 (4)	0 (0)	26 (96)	27
YouTube	1 (4)	1 (4)	22 (92)	24
Other	1 (10)	2 (20)	7 (70)	10

**Table 5:** Social media platforms considered to be most useful when undertaking CPD ranked from 1 (extremely useful) to 5 (not useful).

Usefulness for CPD	Extremely useful 1	2	3	4	5 not useful	Total
	Frequency (%)	Frequency (%)	Frequency (%)	Frequency (%)	Frequency (%)	Frequency
Facebook	5 (8)	14 (23)	16 (26)	14 (23)	12 (20)	61
Instagram	0 (0)	2 (4)	9 (18)	10 (19)	30 (59)	51
Twitter	2 (4)	8 (17)	5 (11)	6 (13)	26 (55)	47
LinkedIn	5 (10)	6 (12)	9 (19)	3 (6)	26 (53)	49
ResearchGate	4 (8)	6 (13)	7 (15)	1 (2)	30 (62)	48
You‐Tube	7 (13)	8 (15)	13 (25)	7 (13)	18 (34)	53
Other	1 (12)	0 (0)	3 (38)	1 (12)	3 (38)	8

Participants were asked about their experience with online professional behaviour as well as their reasons for using social media for CPD. When describing online professional behaviour, no participants that use social media platforms for CPD found online behaviour to be unprofessional, 59% (*n* = 41) of participants indicated online behaviour to be professional, and 41% (*n* = 28) selected neither professional nor unprofessional. The most common uses of social media for CPD were following professional organisation pages (54%, *n* = 62), connecting with other MRPs (39%, *n* = 45), connecting with other health professionals (26%, *n* = 30) and using social media to record CPD points (9%, *n* = 10). The most common reasons for social media use were accessibility to information (85%, *n* = 56) little geographical limitations (77%, *n* = 51) and ease of use (74%, *n* = 49). Participants who do not use social media to connect with other professionals (*n* = 45) provided reasons such as that they do not like to mix their professional and private life (51%, *n* = 23), do not have time (38%, *n* = 17) or have privacy concerns (35%, *n* = 16).

### Value of collaborative learning tools

A Friedman test was performed to obtain the overall ranking of importance MRPs attribute to collaborative learning tool for CPD (Table 6). There was a significant difference in ranked importance for all participants (χ^2^ = 242.991, df = 4, *P* < 0.001), as well as participants from rural and remote workplaces (χ^2^ = 45.491, df = 4, *P* < 0.001). Post hoc pairwise comparison adjusted by Bonferroni correction for multiple tests indicated that the difference in ranking between seminars, workshops and conferences was not statistically significant for all participants (*P* > 0.05) as well as those from rural and remote workplaces (*P* > 0.05). In contrast, difference in ranking for social media groups and journal clubs with seminars (*P* < 0.001, *P* < 0.001), workshops (*P* < 0.001, *P* < 0.001) and conferences (*P* < 0.001, *P* < 0.001), respectively, was statistically significant, with the exception of social media group conferences for practitioners from rural and remote locations (*P* = 0.083).

**Table 6:** Order ranking of importance of collaborative learning for CPD (5 very important to 1 not important) for the overall population (*n* = 115) compared with those working in a rural or remote location (*n* = 25).

Collaborative learning tool	Mean value of the participants	Mean value rural and remote
Seminars	3.83	3.80
Workshops	3.75	3.74
Conferences	3.67	3.44
Journal club	2.01	1.76
Social media groups	1.73	2.26

### Workplace support for CPD

The majority of respondents (80%, *n* = 92) reported they were able to undertake CPD during work hours either through dedicated time allocation (49%, *n* = 56) or in an unofficial capacity (31%, *n* = 36). Difference in dedicated time allocation across health sector was evident (Fisher’s exact test = 9.121, *P* = 0.021) with 58% (*n* = 41) of participants working within the public health sector allocated dedicated time for CPD, compared to 33% (*n* = 14) in the private sector. Geographic location also was associated with the provision of dedicated time for CPD during work hours (Fisher’s exact test = 13.498, *P* = 0.025) with MRPs in metropolitan (57%, *n* = 41) and regional (53%, *n* = 9) workplaces afforded this benefit compared with their colleagues employed in rural or remote locations (23%, *n* = 6).

Workplaces can also support MRP attendance at conferences through the provision of financial support and leave. Most workplaces were observed to provide funding and paid leave for conferences if staff members were presenting or attending (Table 7). Only 20% (*n* = 21) have all or the majority of their travel expenses paid for by their workplace, and 29% (*n* = 30) have no travel paid. A percentage (13–20%) of participants were unsure of workplace supports which may be available to them to attend conferences.

**Table 7:** Workplace support to attend conferences (*n* = 103).

Workplace support for conferences		Frequency (%)
Funding	Yes, if presenting	39 (38)
	Yes, if attending	52 (50)
	No	14 (14)
	Unsure	19 (18)
Leave	Yes, paid leave if I am presenting	35 (34)
	Yes, paid leave if I am attending	63 (61)
	Yes, unpaid leave if I am presenting	4 (4)
	Yes, unpaid leave if I am attending	10 (10)
	No	11 (11)
	Unsure	20 (20)
Travel expenses	Yes, all travel expenses are funded	6 (6)
	Majority of travel expenses	15 (14)
	Some travel expenses	39 (38)
	No funding for travel expenses	30 (29)
	Unsure	13 (13)

Percentages are based upon the total participants responded to this question; participants can select multiple answers.

## Discussion

Demographic data were used to determine the representativeness of the sample population to the Australian population of registered MRPs.^18^ In this study, 70% of respondents were female, which is comparable to national MRP data (68.1%).^18^ The age of participants in this study (Table 1) is similar to those reported for Australian MRPs; for example, 33% are 30–39 years and 20% 40–49 years.^18^ In relation to area of specialisation, survey respondents (Table 1) are similar to national MRP data of 78% registered diagnostic radiographers, 15% radiation therapists and 7% are nuclear medicine technologists.^18^ These findings suggest that the sample is representative of Australian MRPs.

Participating in workplace training, online learning, reading articles and attending a workplace seminar were the most commonly used CPD activities in this study (Table 2). These findings are similar to Sholer et al.^19^ who reported that the most common CPD activities utilised among Western Australian radiographers were information sessions initiated by their employers, supervising students, reading scholarly literature and attending ASMIRT run courses and seminars. This current study has shown that MRPs in metropolitan locations attend workplace seminars (*P* = 0.025) and conferences (Table 3) more regularly than their colleagues employed in rural or remote locations. Lee et al.^5^ identified that MRPs working in rural areas lack access to CPD activities such as attending a course and suggested that the use of online resources could be beneficial in this circumstance. This current study has established that there is greater use of online learning for CPD by rural and remote MRPs than their regional and metropolitan colleagues (*P* = 0.012; Table 2). This suggests that MRPs in rural or remote areas are utilising online learning more frequently to counterbalance the lack of access to other CPD activities such as seminars and conferences.

More than half of the MRPs in this study participate in online learning using social media. Facebook was most frequently accessed and considered the most useful platform for CPD, while LinkedIn and Instagram were used less frequently on a monthly basis for CPD (Tables 4 and 5). Boyd et al.^11^ investigated online media use for CPD among Australian and Canadian MRPs and identified YouTube was accessed most frequently (approx. 28% of respondents) followed by Facebook (approx. 20%). In addition, Boyd et al.^11^ reported that MRPs expected their use of YouTube to increase (to 35%) in the next 12 months and their use of Facebook to decrease to 15%. This current study demonstrated a higher overall frequency of the use of Facebook (*n* = 51, 44% of all (115) respondents) for CPD and lower use of YouTube (*n* = 24, 21% of all respondents) than that of Boyd et al.^11^ MRPs recognise that credibility of online information is highly important and is a reason why some social media resources are less useful for CPD.^11^ As MRPs in this current study were using Facebook to follow professional organisation pages and to connect with other MRPs and health professionals, this suggests they are using social medial to access credible sources of information for professional learning.

The high ranking of seminars and conferences observed in this current study accords with previous research^6^ and may reflect both the opportunities they provide to participants to interact and share knowledge with others as well as being the traditional initial routes for dissemination of new knowledge within professions.^20^ The high ranking of seminars and conferences also highlights the importance that these traditional face‐to‐face educational tools continue to have for learning in the 21st century. MRPs in rural or remote geographical locations similarly placed higher importance ranking to seminars, workshops and conferences for CPD, with an increase in ranked importance of social media groups observed (Table 6). This signifies the role that social media may play when other preferred face‐to‐face educational tools are not readily accessible. This presents an ongoing opportunity for individuals and organisations to consider innovative methods in providing rural and remote practitioners with remote access to highly valued workshops, seminars and conferences for CPD. With COVID‐19 resulting in an increased use of remote (virtual) learning for seminars and training workshops (e.g. IV cannulation and contrast media workshops offered through ASMIRT),^21^ it would be beneficial to identify whether the use of remote learning becomes more established and highly valued for CPD in the future.

Over half of the participants within this study were officially or unofficially allocated time for CPD activities during work hours (Table 7). MRPs within the public health sector were provided more dedicated CPD time than their colleagues within the private sector. This finding is in accordance with Shanahan et al.^22^ who reported that more MRPs employed in the public sector (35%) were provided with time for professional reading by their workplace than their colleagues in the private sector (14.3%, *P* < 0.001). Previous studies have also identified that funding and cost are major barriers to undertaking certain CPD activities.^5^, ^10^, ^19^ Results within this current study identified that participants within the public health sector are more likely to be provided with financial support and paid leave if presenting or attending a conference compared to those within the private sector (Table 7). This discrepancy in support for CPD across workplaces may impact the professional development of MRPs and ultimately the quality of patient care. In addition, as there were a number of participants unsure of financial and leave supports provided by their organisation for CPD, workplaces should ensure employees are aware of supports available to them to facilitate their professional learning.

Geographic location can influence access to learning opportunities. The results from this current study identified that MRPs in rural or remote locations receive less funding, paid leave and travel expenses to attend conferences than those in metropolitan locations. Lee et al.^5^ ascertained that access was the major barrier for MRPs in rural locations. MRPs in rural locations were required to travel further resulting in increased travel expenses and leave difficulties as there was less staff available for leave cover.^5^ This current study confirms that these barriers of time, financial support and geographic access remain and these are recognised as common deterrents to attending conferences and face‐to‐face collaborative learning activities.^5^, ^9^, ^10^

The majority of respondents using social media for CPD in this current study reported that social media provided easy access to information, was considered to be time efficient and low cost with no limitations such as geographical location. Using social media as it is less time‐consuming has previously been reported.^2^ Greater use of social media for CPD could alleviate some of the barriers identified for face‐to‐face learning such as geographic location and cost.^11^, ^14^ Not all MRPs are comfortable using social media for CPD^11^ with concerns regarding online privacy, online behaviour and a preference to keep their private and personal life separate. Participants in this current study who did not use social media for CPD (*n* = 45) also identified that they do not like to mix their professional and private life and had privacy concerns. Despite these noted barriers, the majority of respondents reported that when using social media for CPD purposes, the online behaviour encountered was professional.

It is recognised that the value of collaborative learning tools identified in this study may have changed dramatically due to COVID‐19. Face‐to‐face collaborative learning has been more restricted and as a result online or remote learning has become widely adopted.^21^ It is therefore recommended that a follow‐up study be undertaken to determine the impact of COVID‐19 on frequency and value of collaborative learning tools for CPD.

### Limitations

A number of limitations are associated with this study. Firstly, sample size was small (*n* = 115), resulting in a 9% margin of error.^17^ As such, survey results must be interpreted with caution. Secondly, this study relied on convenience sampling where respondents volunteered to participate via an email blast with the questionnaire invitation link. This method of dissemination is particularly vulnerable to sampling bias as those who utilise technology are more inclined to participate.^23^ As MRPs are required to continually update their knowledge regarding technology, this inherent requirement of the profession may reduce the impact of sampling bias. Thirdly, the educational tools examined in this study were limited. As such, survey results must be interpreted within the framework of educational tools investigated.

## Conclusion

Medical radiation practitioners currently utilise both face‐to‐face and online collaborative learning tools to meet their CPD needs. Face‐to‐face collaborative learning tools are highly utilised and valued by MRPs. Barriers including time, funding and location can be a deterrent to participation in face‐to‐face learning. Social media platforms for CPD can potentially eliminate these barriers providing easy to access information regardless of geographical location and connection with other professionals. While CPD is an individual requirement for registration,^3^ the benefits are harnessed within workplaces with improved patient care and safety.^3^ Organisations must have knowledge of and address the issues that currently limit CPD opportunities for MRPs so that they can stay up to date with the changing knowledge base of their profession and provide high‐quality evidence‐based care to their patients.

## Conflict of Interest

The authors declare no conflict of interest.
